# Acknowledgment to Reviewers of *Life* in 2021

**DOI:** 10.3390/life12020199

**Published:** 2022-01-28

**Authors:** Life Editorial Office

**Affiliations:** MDPI AG, St. Alban-Anlage 66, 4052 Basel, Switzerland

Rigorous peer-reviews are the basis of high-quality academic publishing. Thanks to the great efforts of our reviewers, *Life* was able to maintain its standards for the high quality of its published papers. Thanks to the contribution of our reviewers, in 2021, the median time to first decision was 15 days and the median time to publication was 35 days. The editors would like to extend their gratitude and recognition to the following reviewers for their precious time and dedication, regardless of whether the papers they reviewed were finally published:
Abate, AndreaLeirós-Rodríguez, RaquelAbbasi, NaghmehLenzini, LiviaAbbey, DeeptiLeón, FranciscoAbd El Maksoud, Ahmed I.Leoni, Matteo Luigi GiuseppeAbdelaziz, MarwaŁepecka, AnnaAbdelgied, MohamedLerma, ClaudiaAbd-Elsalam, KamelLeroy, BaptisteAbidi, Syed HaniLesyk, RomanAbomoelak, BassamLeszczynska, KatarzynaAbraham, RachyLeung, Theresa Ngan HoAbu-Rumeileh, SamirLevchenko, VladimirAbuyassin, Bisher H.Lewandowski, Łukasz B.Acedo, JeellaLi, Chia-JungAcevedo, RogelioLi, Chia-YangAci-Sèche, SamiaLi, Chien-FengAcosta-Avalos, DanielLi, GuangyuAdami, Guy R.Li, HeAdanero Velasco, AlbertoLi, HongjunAdhikari, ManishLi, JianAdhikari, PrakashLi, JieAdhikari, Rajan DeepLi, LiliAdolf-Bryfogle, JaredLi, NingAfanasieva, OlgaLi, WeiAgakidis, CharalamposLian, TengxiangAgarwal, PoonamLiapi, CharisAgresta, FerdinandoLichtenauer, MichaelAguayo Paul, SebastiánLiebman, MichaelAhamad, ShahzaibLievens, AntoonAhmad, Baseer U.Lighezan, DanielAhmad, FahimLim, Han KyuAhsan, Mohamed JawedLim, Si-keunAiroldi, GiorgioLima, Lucas KennedyAjduk, AnnaLin, Chang-ShenAkashi, MasayaLin, Chien-yuAkhavan-Sigari, RezaLin, Chih-ChingAkkouch, AdilLin, Chin-HsienAksan, AysegülLin, Chin-YuAlam, Mohammad ShahLin, Chuen-FuAlba, JosephineLin, Han-JiaAlbarillo, Fritzie S.Lin, Hsiao-HsienAlbuquerque, CyroLin, Hsing-YingAlcántara-Ortigoza, Miguel AngelLindahl, LasseAleksić, IvanaLindberg, GabriellaAlexandra Stanescu, Ana MariaLindgren, AnttiAlexandrova, AlinaLingam, GopalAl-Gharaibeh, AbeerLingam, ManasviAlhadrami, HaniLipert, AnnaAliev, RamizLis, MarcinAlizargar, JavadLittle, JulianAlkelai, AnnaLiu, EnqiAlkhamis, Mohammad ALiu, HengruiAlmagro Armenteros, Jose JuanLiu, NingAlmeida, DanielaLiu, QingboAlok, AnshuLiu, YongfengAlonso-Colmenar, Luis M.Liwo, AdamAlqahtani, JaberLlanes-Álvarez, CarlosAlsaad, Saad M.Lo Cascio, GiulianaAl-Shargie, FaresLobo, JoaoAltamura, EmilianoLocascio, AntonellaAltmann, BrigitteLocatelli, DavideAltwegg, KathrinLocatelli, LauraAluri, JahnaviLoffredo, LorenzoÁlvarez-Rodríguez, ManuelLok, Chun NamAly, HanyLok, Hiu ChuenAmalfitano, StefanoLokesh, Belur RAmarelli, CristianoLopes Ferreira, MonicaAmber, Kyle T.López Garcia, José ManuelAmedeo, BalbiLopez, JoelleAmeta, SandeepLópez, LeonardoAmiri-Yekta, AmirLópez-Campos, FernandoAmoresano, AngelaLópez-Fernández, JorgeAn, Hyo JinLópez-Hernández, Luz BereniceAnagnostopoulos, IoannisLopez-Picado, AmandaAnand, KartikLópez-Pintor Muñoz, Rosa MaríaAnand, SachitLópez-Plaza, DanielAndo, IstvanLopez-Reyes, GuillermoAndrade, José PedroLoscocco, Giuseppe G.Andree, Karl B.Losi, Maria AngelaAngelopoulou, Matina V.Loughrey, DavidAnghel, LarisaLoukovitis, DimitriosAngriman, ImerioLovisa, FedericaAnnaratone, LauraLowe-Krentz, LindaAnnenkova, Nataliia V.Loy, FrancescoAntičević, DarkoLu, LingengAntonakopoulos, NikolaosLu, QuanlongAntonucci, SalvatoreLu, TaoAntony, Justin SLu, ZhiyingAntunes, JorgeŁuc, MateuszAoki, MakotoLucà, FabianaAouissi, Hani AmirLuca, GiovanniAparicio, InmaculadaLucarini, GuendalinaApone, FabioLucattelli, MonicaAppel, JensLucchetti, DonatellaAppenzeller, SimoneLuciani, MichelangeloArad, MichaelLuciani, MirellaAragón Vela, JerónimoLucien, FabriceAraj, Faris G.Ludzik, JoannaArantes, Pablo RicardoLugović-Mihić, LiborijaAraújo, MárioLunardi, MicheleArboix, AdriàLuo, QunArbune, ManuelaŁupicka-Słowik, AgnieszkaAresta, AntonellaLushington, GerryArgo, AntoninaLutfi, EsmailAriani, AlaricoLütz, StephanAriyawansa, HiranLyck, RuthArnoldt-Schmitt, BirgitMa, Chih-MingArora, KomalMa, DuanArvanitis, DemetriosMa, FengyunAsdaq, Syed M.B.Macarthur, DonaldAshkenasy, GonenMacDonald, AnitaAshley, Ryan L.Machado, Denis JacobAspinall, MichaelMachairas, NikolaosAste, GiovanniMacias, AlvaroAthanasiou, Labrini V.Macica, Carolyn M.Atlante, AnnaMaciejewski, RyszardAudisio, Marcela CarinaMaciver, SutherlandAuer, Kerstin E.Mackowiak, MarzenaAugustine, JosyMadigan, Michael T.Augusto, LeonardoMadsen, NielsAujayeb, AvinashMaeda, KazuhikoAunapuu, MarinaMaeda, NoriakiAustin, ShaneMagalhães, JoanaAvanzas, PabloMagalhães, LuísaAvilla, LeonardoMagiorkinis, Emmanouil N.Avishai, GalMaglio, AngelantonioAvram, Mihaela FlaviaMagnan, BrunoAzarin, Kirill VitalievichMagrì, AndreaAzcona San Julian, María CristinaMahant, NeilAzevedo Santos, HuarrissonMahmoud, Eman E.Azevedo, ElsaMahnert, AlexanderAziz, FaisalMaia, CláudioAzziz, RicardoMainra, RahulBabkin, Igor V.Mair, Lamar O.Babu, Gopal J.Maiti, SampaBadau, AdelaMaj, MalgorzataBadescu, Minerva CodrutaMajumder, KinjalBae, YoungsookMak, Gannon C. K.Baez, María VerónicaMäkelä, Juho AnttiBagacean, CristinaMakin, StephenBagci, SoyhanMakiyan, ZograbBagetta, GiacintoMalar, Mathu C.Bagiu, Iulia-CristinaMalcangi, GiuseppinaBahmad, HishamMałecki, PawełBai, GaowaMalinowska-Lipień, IwonaBai, ShijieMalińska, DominikaBaig, Mohammad HassanMalkovsky, Victor IoannovichBajaj, JitinMallamaci, RosannaBala, SaikatMalmendal, AndersBalakirev, Evgeniy S.Maloberti, AlessandroBalan, ShabeeshMalysh, Julia M.Balasch, MonicaMałysz-Cymborska, IzabelaBaldassano, SaraMancia, AnnalauraBalieva, Flora N.Mancini, CeciliaBaliti, JamalMancini, InesBalla, AndrásManco, GiuseppeBallarò, RiccardoMandal, AmritlalBallaz, Santiago J.Mandala, Maria TeresaBalogh, GáborMandava, NandaBaltuch, Gordon H.Mandler, WilliamBaltzinger, ChristopheManelis, AnnaBandow, KenjiroManfredini, MarcoBanf, MichaelMangel, László CsabaBanskota, SuhridMangiaterra, GianmarcoBánvölgyi, AndrásManhart, MichaelBaranowska-Wasilewska, MarlenaMani, MuralidharanBarański, KamilManier, Sascha K.Barbagallo, FedericaManolescu, Loredana Sabina CorneliaBarbara, BreznikManousi, NataliaBarberio, BrigidaMansilla, Juan RodríguezBarbes, LucicaMantero, MarcoBarbieri, RobertoManuel-Apolinar, L.Barbier-Torres, LucíaManukumar, H.M.Barghout, SamirMaoz-Segal, RamitBargiela, AriadnaMapuskar, Kranti A.Baron, ByronMaranesi, ElviraBaroncini, AliceMarasca, FedericaBaroni, StefanoMarchi, GiacomoBarrea, LuigiMarcoleta, Andrés E.Barreto, GoncaloMarconi, Anna MariaBarros-Barbosa, Aurora RaquelMarcu, LoredanaBarrott, JaredMargalida, AntoniBartas, MartinMarginǎ, DenisaBarten, Markus JohannesMargolis, Amy E.Bartnik, EwaMárialigeti, KárolyBartolomeo, NicolaMariet, Anne-SophieBasdeo, ShareeMarina, DiotalleviBatista, RamonMarini, AlessandroBattafarano, Daniel F.Marini, Herbert RyanBazsalovicsová, EvaMarinkovic, BratislavBeare, NicholasMarkopoulos, Georgios SBeck, SarahMaroneze, MarianaBecker, SidneyMarongiu, LuigiBędkowska, Grażyna EwaMarotta, NicolaBehar, AlbertoMarques, DuarteBehnke-Borowczyk, JolantaMarques, Joana A.Behringer, ErikMarquetand, JustusBelinchón, IsabelMarsili, LucaBelinky, FridaMartin, FranckBellé, CristianoMartin, OlgaBellezza, IlariaMartin, SteggallBellinato, FrancescoMartina Battaglia, AnnaBellucci, LucaMartina, SandonáBelotserkovskaya, RimmaMartínez De Morentin, Pablo BlancoBelov, GeorgeMartínez-Gil, NataliaBenabdelkamel, HichamMartínez-Laperche, CarolinaBenedetti, AntonioMartínez-Lara, EstherBenitez-Temiño, BeatrizMartínez-Nova, AlfonsoBennardo, LuigiMartínez-Pérez, OscarBennett, AnnemarieMartinotti, GiovanniBennett, Christopher J.Martinotti, SimonaBenoit, CournoyerMartins, Ana D.Bensch, KonstanzeMaruzzo, MarcoBentivegna, EnricoMarverti, GaetanoBentley, DavidMarzoli, FrancescaBentley, Robert F.Mascetti, JoëlleBenzaoui, AmirMascitti, MarcoBeola, LilianneMäser, PascalBepari, Asim KumarMashima, IzumiBéraud-Dufour, SophieMasselli, ElenaBerchialla, PaolaMassimiani, MicolBerezin, AlexanderMastroleo, FedericoBerg, JohannaMastropietro, AlfonsoBergamini, ChristianMasuda, MariBergantini, LauraMateus, CeuBermon, StephaneMathis, ColeBernardeschi, DanieleMatić, AnitaBernstein, NiritMatikonda, SiddharthBerry, BrentMatsubara, ShigekiBerry, Irina MaljkovicMatsuhisa, FumikazuBerthon, Annabel S.Matsumoto, KazumasaBertini, FlavioMatsushima, TakashiBertini, LauraMatulić, MajaBertoglio, DanieleMatveeva, MariiaBesedovsky, LucianaMatveeva, TatianaBester, ChristoMaugeri, AlessandroBestwick, MeganMaurel, Marie-ChristineBeszédes, SándorMaurelli, MartinaBeuf, OlivierMaury, Carl Peter J.Bevc, SebastjanMavrogonatou, EleniBharadwaj, ShivMay, Christian AlbrechtBhattacharya, SomanonMazzone, PellegrinoBhutta, Mahmood FazalMazzoni, MariaBiałynicki-Birula, RafałMcCarthy, RayBianchi, Luca Nicola CesareMcCombie, AndrewBianucci, GiovanniMcGinnis, Graham RBikov, AndrasMcGregor, GlennBikov, AndrásMcminn, AndrewBillon, CyrielleMeans, Robert T.Binkowski, ŁukaszMedian, DragosBirsa, Mihail LucianMehdi, SyedBirzer, CristianMéhes, GáborBivona, GiuliaMehta, Nihar J.Bjelic, SinisaMehta, SanjayBjørkavoll-Bergseth, Magnus FriestadMeijsing, SebastiaanBjörn, Lars-OlofMeimandi, Kiana JafariBlachowicz, AdrianaMeka, Durga PraveenBlack, RoyMelo, Pedro L.Blackburn, JessicaMendoza-Nuñez, Victor ManuelBlagojevic, DuskoMenéndez-Arias, LuisBlanton, CynthiaMenezes, Miriam-RoseBlasdell, KimMeng, QinghangBlasiak, JanuszMerah, OthmaneBo, MarcoMerchant, MichaelBoarescu, Paul-MihaiMercury, Santo RaffaeleBoaventura, PaulaMerkulyeva, NataliaBobkov, DanilaMeroni, GabrielleBocchieri, SalvatoreMeroni, PierluigiBöckmann, AnjaMesa, FranciscoBoda, FranciscMessineo, DanielaBoddupalli, AnuraagMester, AndraBoffano, MicheleMettler, Lieselotte L.Bogen, JanMetzinger, LaurentBogers, Willy M J MMeunier, FredericBohmwald, KarenMhatre, SiddhitaBoiani, MicheleMichalak-Stoma, AnnaBolla, Pradeep KumarMichalis, EurydikiBonaccorsi Di Patti, Maria CarmelaMichels, AlexanderBonanad, SantiagoMichielsen, PeterBonciu, ElenaMichita, Rafael TomoyaBondurand, NadegeMiękus, NataliaBonfio, ClaudiaMiele, RossellaBoni, RaffaeleMier, PabloBono, NinaMieszkowski, JanBonucci, AlessioMigliorini, FilippoBoroń, AlicjaMihál, IvanBorowicz, MarcinMilaniak, IrenaBorriello, GiovannaMilano, FrancescoBorzikowsky, ChristophMiles, JohnBose, Rajendran JCMileva, MilkaBoti, Michaela A.Milhano Santos, FátimaBoto, LuisMiličić, DraganaBouchet, AudreyMilkova, EvaBoussios, StergiosMills, James K.Bowen, AnthonyMin, Kyung-JinBozdoğan, HakanMiñana, GemaBozic, JoskoMingot-Castellano, María EvaBozin, BiljanaMinichsdorfer, ChristophBracci, LuisaMinistrini, StefanoBracher, FranzMino, SayakaBrack, AndreMintāle, IvetaBrandt, AdamMiranda, JonatanBrault, JeffreyMiranda, LuisaBrennenstuhl, HeikoMiro, JordiBresciani, RobertoMiroslaw, KiedrowskiBrigandt, IngoMirza, AhmadBrioschi, MarcosMirzaei, SiroosBrkic, Faris F.Misaka, TomofumiBrodosi, LuciaMisawa, AyaBroncel, MarlenaMishra, AwanishBroquet, AlexisMishra, Jay S.Bros, MatthiasMishra, RibhavBrower, AmyMiszczyk, JustynaBrüggmann, DörtheMititelu Tartau, LilianaBrukman, NicolasMitsuyama, SusumuBrundage, Cord M.Miura, TakehsiBrunk, Clifford F.Miyauchi, SeijiBruschini, LucaMizsei, RékaBruzzese, LaurieMizuno, TomohiroBrytek-Matera, AnnaMizuuchi, RyoBrzezinski, AmnonMladenov, EmilBu, LijingMlynarczyk, DariuszBucher, SimonModesto, PaolaBuchkovich, Nicholas J.Modi, JigarBukic, JosipaMognetti, BarbaraBurlacu, AlexandruMohammadi, SaeedBurton, Aaron S.Mohan, GaneshBuschmann, AlejandroMojiri, AminBusetto, Gian MariaMokhtar, HelmyButch, Christopher J.Mokros, ‪ŁukaszButera, AndreaMolero, DavidButler, DavidMolina Salinas, Gloria MaríaBüttner-Teleagă, AntjeMolter, DanielCabana-Domínguez, JuditMolyneux, KarenCabrales, Pedro J.Mondanelli, NicolaCaccamo, DanielaMondejar-Lopez, PedroCacciola, FrancescoMong, Mei-ChinCadossi, RuggeroMonitillo, FrancescoCaeiro, Maria FilomenaMontecucco, FabrizioCaetano-Anollés, GustavoMontero, OlimpioCalabriso, NadiaMontibus, MathildeCaldiroli, AliceMontisci, AndreaCalina, DanielaMonzón, MartaCallejón, RocioMooli, Raja Gopal ReddyCaltabiano, RosarioMor, EytanCalvaruso, MarcoMorales, SerafinCalzadilla, PabloMorales-González, José AntonioCampagna, RobertoMoral-García, José EnriqueCampesi, IlariaMorar, AdrianaCampinho, MarcoMorasiewicz, PiotrCampione, ElenaMoravcikova, JanaCampisi, RaffaeleMoreau, MagaliCancelli, AlexanderMorel, Jean-LucCancian, MauroMorett, EnriqueCanè, StefaniaMoretti, BiagioCangussú, Maria C.T.Morgan, ClaireCanini, FabianaMori, MasaakiCannataro, RobertoMorimoto, KatsuhikoCanuti, MartaMorita, MasamuneCao, HongnanMoro, LoredanaCaparros-Martin, JoséMorouço, PedroCappellini, IacopoMortreux, MarieCaraglia, MichèleMosieniak, GrazynaCaramenti, MartinaMoskvin, SergeyCarbone, FedericoMostafa, ElachouriCardona, FernandoMotamed, CyrusCardoso, Danon ClemesMottet, DenisCardozo-Filho, LúcioMotwani, Hitesh V.Carl, Hans-DieterMotwani, MonaCarmo, IsabelMou, LishaCarnegie, RyanMozas-Moreno, JuanCaron De Fromentel, ClaudeMroczek, AgnieszkaCarraro, MichelaMueller, Christian A.Carriconde, FabianMuhovski, YordanCarullo, GabrieleMullineaux, Conrad W.Caruntu, ConstantinMulloy, BarbaraCasaas, AdrianaMun, Je-HoCasado-Hernández, IsraelMuniraj, NethajiCasal-Guisande, ManuelMunoz, MariaCasari, AliceMuñoz-Pousa, IsabelCasarotto, RaquelMünster-Wandowski, AgnieszkaCascella, RaffaellaMurase, DaikiCassone, GiuseppeMuratani, MasafumiCastellani, DanieleMurdica, ValentinaCastelli, Maria EugeniaMurphy, Patrick B.Castro, RitaMurru, ElisabettaCatalin, BogdanMurugesan, SelvasankarCatanzano, OvidioMusha, AtsushiCavallero, SerenaMytych, JenniferCaviglia, Gian PaoloNabi, NesrineCayre, MyriamNadeau, JayCebola, Maria JoãoNagai, NorihiroCeccarelli, GabrieleNagano, HanatsuCeci, AndreaNagaraju, Shankar PrasadCeci, MarcelloNagayasu, EijiCelakovska, JarmilaNagovitsyn, Roman S.Celenza, GiuseppeNagy, BálintCendrowski, EmilyNair, KalyaniCentonze, LeonardoNair, SmitaCeolotto, GiulioNajmanová, LucieCerri, MatteoNakagawa, HiroshiCervellati, FrancoNakai, KentaCesa, StefaniaNakamura, MasatoshiCesareo, RobertoNakano, MasahitoCetiner, MustafaNakhjavani, MaryamCevolani, LucaNalos, MarekCha, Jung-JoonNaňka, OndřejChalas, RenataNapolitano, FilomenaChan, Chia-Hsin (Lori)Narzisi, AntonioChan, Tze-SianNaserian, SinaChandru, KuhanNaskar, ShovanChang, Ching-JinNassif, Aude SophieChang, HsiNasso, GiuseppeChang, Hsueh-WeiNatale, GianfrancoChang, Jia-MingNathanailides, CosmasChang, Ke-VinNavarrete, PaolaChang, Melinda Y.Navarro Cuéllar, CarlosChang, Nai-JenNavarro, Francisco J.Chang, Wen-WeiNawab, AkbarChappard, ChristineNawaz, Muhammad AmjadChatterjee, SampurnaNazzaro, GianlucaChatterjee, SankarNeagu, Tiberiu-PaulChaube, Bal KrishnaNeerman-Arbez, MargueriteChavan, SameerNegus, DavidChazal, NathalieNeigel, JosephCheetham, Seth W.Németh, Tibor MihályChen, ChunNeophytou, Christiana M.Chen, ChushuangNesterov, DmytroChen, Der-YuanNeubeck, AnnaChen, IdaNewhouse, IanChen, Jin-PingNg, JulieChen, John Jing WeiNg, KenChen, Min-HuaNghe, PhilippeChen, Po-WenNguyen, ScottChen, RuiNicod-Lalonde, MarieChen, Shyi JouNicola, LongoChen, WeichihNicolaidou, ElectraChen, YihaoNicole Theresa, VargasCheng, Chun-AnNicosia, AldoCheng, Hong ShengNieland, John Dirk VestergaardCheng, Juei-TangNielsen, SheilaChi, Chih-YuNiemi, NatalieChiang, Meng-TsanNiethammer, MartinChieffi, PaoloNieto Fontarigo, Juan JoseChifiriuc, Mariana CarmenNighoghossian, NorbertChikina, AleksandraNikkels, Arjen FChin, Tan BoonNikolakis, GeorgiosChinappi, MauroNikolova, GalinaChinen, TakatoshiNimmo, Alan J.Chinnakannan, SenthilNirengi, ShinsukeChiou, Shiow-HerNirmal, JayabalanChirosa Ríos, Luis JavierNischwitz, SandraChiu, MartinaNishida, NorihiroChmielarz, PiotrNishihara, HidenoriChoi, Min SikNishikawa, HirokiCholon, DeborahNitschke, FelixChou, Kuo-ChenNitschke, WolfgangChou, MingchungNitto, Antonio DiChovatiya, Raj J.Noack, SandraChow, Franklin Wang-ngaiNordio, MaurizioChrzanowska, AlicjaNorful, Allison A.Chu, Hee HoNorton, CharlesChua, Eng GuanNorton, ElaineChuffa, Luiz Gustavo De AlmeidaNoureini, Sakineh KazemiChung, Felicia Fei-LeiNovak, IvanaChyau, Charng-CherngNovello, SalvatoreCianci, PasqualeNovosel, DinkoCiani, FrancescaNowak, Michal S.Ciarcia, RobertoNowicka, GrażynaCiccone, ValerioNowomiejska, KatarzynaCicero, ArrigoNozzoli, FrancescoCichero, ElenaNuevo, MichelCichorek, MiroslawaNzwalo, HipolitoCieślińska, AnnaOberle, Doris F.Cifelli, PierangeloOda, HirokiCimmino, GiovanniOgawa, SatoshiCincotti, AlbertoOgier-Denis, EricCiniglia, ClaudiaOgoh, ShigehikoCiocan, CorinaOgórek, RafałCiodaro, FrancescoOğuzoğlu, Tuba ÇiğdemCiorap, RaduOhashi, NamiCipolli, MarcoOhbuchi, ToyoakiCiriaco, FulvioOhl, KimCirillo, GiuseppeOhlendieck, KayCiszkiewicz, AdamOhnuma, MioCiura, KrzesimirOhta, ShinClaudi, RiccardoOhtsuka, SusumuClaudia, ZaniOishi, AkioCocco, RaffaellaOka, AkihikoCoelho, AdrianoOkazaki, YasumasaCohen, MichaelOkovityi, Sergey V.Cohen-Salmon, MartineOku, HidehiroCohuo, SergioOkumura, KoColás, CarlósOkumura, NobuoColasanti, RobertoOlah, MartaCole, Logan W.Oláh, PéterColeman, Anthony WilliamOlaru, Octavian TudorelColeman, ChristopherOlasagasti, FelixCollins, Justin W.Olivier, Jean LucColson, PhilippeOlszewski, Michal AComi, CristoforoOmbrello, Michael J.Comini, LauraO’Neil, NigelConde-Pueyo, NuriaOo, Joo HyunConniot, JoãoOpländer, ChristianConsorti, LorenzoOpp, SilvanaCooper, Arthur J.L.Oppenheim, SaraCoquerel, DavidOrczyk-Pawiłowicz, MagdalenaCoradin, MarielO’Reilly, ShaneCorallo, FrancescoOrlova, AnnaCorbera, Juan AlbertoOrlova, NadezhdaCordie, David R.Orlova, OlgaCoricovac, DorinaOrme, JacobCorrado, GiovanniOrnello, RaffaeleCorral, JavierOrso, EvelynCorral, PaulinaOry, StéphaneCorreia-Branco, AnaOsipov, Sergey PavlovichCortes, Christian.Osório, Natália MeloCossu, PieroOsredkar, JoškoCosta, LucianaOsti, Mattia FalchettoCosta, MargaridaOstrovsky, OlgaCosta, Maria Do CéuOstuni, MarianoCostagliola, AnnaOtero-Espinar, Francisco JavierCota, CarloOtowa, YasunoriCotella, DiegoOtt, Charlie MarkCovantev, SergheiOttoni, Cristiane AngélicaCrespo-Garcia, SergioOu, LiCrinò, Stefano FrancescoOuahabi, AbdeldjalilCrisan, LuminitaOzarowski, MarcinCrocco, PaolinaPace, FabioCrooke, StephenPacholak, AmandaCruz, Juan C.Paciotti, RobertoCruz-López, RicardoPacurari, MaricicaCruz-Ramos, MarlidPacuta, VladimirCsécsei, PéterPączek, SaraCuda, GiovanniPaczesny, ŁukaszCugliari, GiovanniPadi, Sathish K.R.Cugmas, BlažPaduraru, LuminitaCultrera, RosarioPaissoni, CristinaCunningham, Rory P.Palacios-Jaraquemada, José MCurrò, MonicaPałasz, ArturCzernek, JiříPalazón-Bru, AntonioCzerniejewski, PrzemysławPalazzo, AntonioDa Rocha, Simao TeixeiraPallag, AnnamáriaDa Rold, GrazianaPallotti, FrancescoDąbrowska, JoannaPalm, Gottfried J.Dagnino, AlexiesPalumbo, MarcoD’Agostino, Paul M.Pampalakis, GeorgiosDahl, Mathilde BorgPamporaki, ChristinaDahlmann-Noor, AnnegretPan, ChunDaly, AnnePan, Hung-ChuanDamián, Juan PabloPan, Wen-HarnDamiani, ClaudiaPandiyan, MuthuramalingamDamiani, Gianluca R.Panicz, RemigiuszDamiani, GiovanniPanova-Noeva, MarinaDaneel, MiekePantalone, Mattia RusselD’Angelo, FrancescoPantazaka, EvangeliaDangi, TanushreePaola Loguercio, PolosaDanilović, Bojana R.Paolo, AngeliniDaras, GerasimosPapakostas, SpirosD’Ardes, DamianoPappa, AglaiaDas, BiswadipParađiković, NadaDastoli, StefanoParaskevas, George P.Datta, RitwikPardo, BenjaminDavalieva, KatarinaPare, DramaneDay, Alvin LeeParedes, FelipeDayan, MichaelPareek, MananDe Bonis, PasqualeParikh, Nainesh SDe Caro, RaffaeleParis, JuanDe Chiara, LetiziaPark, Hee BokDe La Torre, Jose GarciaPark, HyungjongDe Leeuw, PeterPark, Hyun-sunDe Luca, PaolaPark, JaeyongDe Miglio, Maria RosariaPark, Jong KookDe Pascali, MariarosariaPark, Jung GilDe Paula Rogério, AlexandrePark, JunsooDe Paz Fernández, Jose AntonioPark, Sook-HyunDe Rasmo, DomenicoParodi, EmiliaDe Sanctis, FrancescoParodi, Pier CamilloDe Sanctis, Juan BautistaParr, Jeffrey J.De Santa Pau, Enrique CarrilloParrotta, ElviraDe Servi, StefanoPârvu, MarcelDe Simone, Salvatore GiovanniPascual, GloriaDe Vos, JohnPasqualone, AntonellaDe Ziegler, DominiquePassalacqua, KarlaDea-Ayuela, María AuxiliadoraPasternak, GrzegorzDeb, SubrataPatel, Devang M.Dębowski, MarcinPatel, Girijesh KumarDeCaro, CarmenPaternoster, GianlucaDeftu, Alexandru-FlorianPatrício, PaulaDegl’innocenti, DonatellaPatsinakidis, NikolaosDegos, BertrandPaul, Amber M.Deigner, Hans-PeterPaulino-Lima, Ivan GláucioDeive, FranciscoPavlović, NebojsaDel Gallo Di Roccagiovine, Maria MaddalenaPawlik, AndrzejDel Grosso, AmbraPawlik, AnnaDel Real, GustavoPease, AnnaDelaspre, FabienPelidou, Sygkliti-HenriettaDelbac, LionelPemp, BertholdDeligiannis, AsteriosPen, Joeri J.Delmonico, LucasPeña Quintana, LuisDeloch, LisaPeña-Amaro, JoséDemarchi, Marco StefanoPeña-Oyarzún, DanielDembiński, ŁukaszPeng, QiDemonacos, ConstantinosPeraza-Jiménez, KarlaDemyda, SebastianPereira, HenriqueDenecke, ShanePerepechaeva, Maria L.Dera, GuillaumePerez, GermanDervenis, PanagiotisPerez, RonenDesguin, BenoîtPergolizzi, SimonaDevy, JeromePerin, PaoloDey, PriyankarPerkovic, Matea NikolacDhiman, KunalPerner, FlorianDi Bernardo, GiovanniPerrone, MariasoleDi Domizio, JeremyPerrotta, AdolfoDi Donato, PaolaPeserico, AlessiaDi Gennaro, FrancescoPešl, MartinDi Giammartino, Dafne CampigliPesta, DominikDi Giulio, MassimoPestana, ManuelDi Liello, RaimondoPetkowski, Janusz J.Di Maria, EmilioPetráš, MarekDi Nora, ConcettaPetrella, CarlaDi Pietro, NataliaPetrotos, KonstantinosDi Silvestri, Claudia AngelaPetrova, BoryanaDi Stefano, LuisaPetruczynik, AnnaDiakou, AnastasiaPetta, IoannaDias, Fernanda F. G.Peyré-Tartaruga, Leonardo AlexandreDias, GuilhermePezone, AntonioDiaz, ArielPezzoli, LauraDíaz-Flores, LucioPfaller, MichaelDíaz-Godínez, GerardoPfuhl, GeritDietl, PaulPhalak, PoonamDimitriadis, KyriakosPiccirillo, EnricoDindelegan, George CalinPiechota-Polanczyk, AleksandraDiniz, InêsPilalas, DimitriosDireito, RosaPilarska, Agnieszka A.Dišlers, AndrisPini, Luigi AlbertoDistl, OttmarPinilla, IsabelDivino-Filho, JosèPinto Da Silva, LuísDługosz-Lisiecka, MagdalenaPinto, ErnaniDmello, CrismitaPiperi, ChristinaDobrowolski, PiotrPiquero-Casals, JaimeDocea, AncaPiri, RezaDoerig, ChristianPiszczatowski, SzczepanDoglio, AlainPivić, RadmilaDomínguez Medina, EduardoPiwowarczyk, KatarzynaDomino, MalgorzataPizzo, ElioDonadu, MatthewPlacek, KatarzynaDonald, HendersonPlachá, IvetaDonatella, DianaPlanas-Iglesias, JoanDong, PengfeiPlatella, ChiaraDordevic, DaniPlatta, HaraldDorenkamp, MarcPlazzi, FedericoDoroftei, BogdanPlesa, AncaDougherty, LauraPlotnikov, EgorDouliez, Jean-PaulPoddighe, DimitriDrabowicz, JozefPodlacha, MagdalenaDragoni, SilviaPoglajen, GregorDragovich, BrankoPohóczky, KrisztinaDrozak, JakubPoje, GorazdDuarte, LiliannePolčic, PeterDubois-Deruy, EmiliePolesskaya, Oksana O.Dubol, ManonPolge, CécileDucza, EszterPolidori, GuillaumeDuda, MałgorzataPollastro, FedericaDuez, CatherinePolvani, SimoneDulskas, AudriusPolz-Dacewicz, MałgorzataDuranti, GuglielmoPonder, AlicjaDurham, Benjamin H.Ponraj, PrabakaranDuskey, Jason ThomasPoot, MartinDutta, PranabanandaPop, Raluca MariaDux, LászlóPopova, Alexandra A.Dvorakova, Magdalena ChottovaPopova, PolinaDvoretsky, Alexander G.Popow, ChristianDyakonov, VladimirPorcu, EleonoraDylag, MariuszPortugal, CamilaDzhalilova, D. ShPosada-Quintero, Hugo FernandoDziemba, OliverPosautz, AnnikaEastman, AlisonPotaczek, Daniel P.Edamakanti, ChandrakanthPothirat, ChaicharnEden, AmirPouzoulet, FrédéricEdgar, JuliaPozzi, CarloEdgeworth, Jonathan D.Pradhan, Bhola ShankarEdler von Eyben, FinnPradhan, MadhulikaEdwards, StephenPramanik, DibyajyotiEftimie, RalucaPrasuhn, JannikEid, NabilPratsinis, HarrisEl Khalloufi, FatimaPressyanov, DobromirEl-Ansary, HosamPrice, Michael JamesElas, MartynaPrimavera, RositaElbadawi, MoePrimorac, DraganElbestawy, AhmedPrintza, AthanasiaElchaninov, AndreyPritesh, JainElias Odusseas, TzavellasPross, AddyElias, Sjoerd GeertProvenzano, AldesiaElsässer, SimonProvenzano, EugenioElsayad, KhaledPrudhon, Jean-LouisElsner, MatthiasPruhova, StepankaElworthy, StonePrusinkiewicz, MartinEmmanouilidou, EvangeliaPryzdial, EdwardEndre Károly, KristófPrzybył, JarosławEngstrom, PatrikPrzybyło, MałgorzataEnkvist, ErkiPrzystupski, DawidEnoki, YukiPtaszynska, AnetaEnomoto, HirayukiPucciarelli, SandraErdem, SelcukPucker, BoasEriani, GilbertPugliese, MariagabriellaErmolaeva, SvetlanaPuigdomènech, PereErnstberger, AntonioPunia, SnehErrichiello, EdoardoPurge, PriitEscriva, HectorPurita, JosephEslam, MohammedPurkamo, LottaEsmaelizad, MajidPushchina, EvgeniyaEspadas Villanueva, IsabelPuslecki, MateuszEspinosa, EmmaPutowski, LechosławEsposito, PasqualePyott, S.J. (Sonja)Esteves, Ana RaquelQian, ChengminEstrada, Marta FQiao, XuEtminani, KobraQuadri, MarikaEufemi, MargheritaQuak, JasperEverest-Dass, ArunQuijada, LuisExindari, MariaQuindry, JohnFabbri, MatteoRábago, DanielFabiana Fernandes, BressanRabien, AnjaFages, AntoineRaco, AntoninoFagone, PaoloRaczkiewicz, DorotaFaivre-Sarrailh, CatherineRadan, MilaFalsini, BenedettoRadowsky, Jason S.Famà, FaustoRafiad, IslamFancello, FrancescoRaghavendra, AksharaFanetti, GiuseppeRagino, Yuliya I.Farah, NairouzRagnini-Wilson, AntonellaFardeau, Marie-LaureRaguse, Jan-DirkFargen, Kyle MichaelRahaman, Md. MizanurFarobie, ObieRahman, Md JuberFarooqi, Ammad AhmadRai, GaneshFarr, Ryan J.Raja, KalpanaFayssoil, AbdallahRajak, Kaushal KishorFeichtinger, MichaelRajput, VishnuFeinman, RichardRallidis, Loukianos SFélix, Luís M.Ramachandran, MohanrajFelli, Maria PiaRamakrishnan, ChandraFeng, CuijieRamalingam, SathishkumarFeraco, PaolaRamazanov, BulatFerenčaković, MajaRamos-Rodríguez, Juan JoséFerguson, GayleRao, Gundu H.R.Fernandes Da Silva, SandroRao, GuodongFernandes Fagundes, NathaliaRapf, RebeccaFernandez, Bernadette O.Rapizzi, ElenaFernández-Ferreiro, AnxoRapone, BiagioFernandez-Lafuente, RobertoRaspor, MartinFernández-Moreno, Miguel ÁngelRathinasabapathy, AnandharajanFernandez-Vizarra, ErikaRausch, PhilippFerragut, CarmenRaz, AeyalFerraz, Maria PiaRazvan-Cosmin, PetcaFerrazzano, GinaRecalcati, SebastianoFerreira, Manuel MarquesRedfern, JamesFerreira, MartinsRedrejo Rodriguez, ModestoFerreira, RodrigoRedwan, Elrashdy M.Ferro, Emer SuavinhoReed, NicholasFiems, DieterReich, AdamFigarella, KatherineReiche, Edna Maria VissociFiliou, MichaelaReiff, TobiasFilipovic, NatalijaReinehr, SabrinaFilippetti, MirkoReischl, BernhardFilippidou, SevastiReka Anna, VassFilippou, Panagiota S.Relic, BiserkaFina, EmanuelaReshkin, Stephan JoelFinelli, CarmineRety, StephaneFingerhut, RalphReuss, FriederikeFionda, BrunoReuter, KlausFischer, FlorianRexer, Karl-HeinzFišer, CeneReyes, MontserratFisk, John D.Rezaei, ElhamFiszer, AgnieszkaReznik, Jacqueline E.Fitschen-Oestern, StefanieRezus, CiprianFitter, JörgRhee, Chin KookFitzhugh, CourtneyRhee, Je-KeunFlajšhans, MartinRibaldone, Davide GiuseppeFlavia Alessandra, GuarnierRiboldi, Giulietta M.Flávio Protásio, VerasRiboldi, IlariaFlemming, SvenRicchetti, FrancescoFlorek, EwaRicci, ClaudiaFlórez, ÁngelesRiccò, MatteoFocosi, DanieleRichard, DaveFogacci, FedericaRichards, Stephen JFoliaki, SimoteRichetta, ClémenceFondevila-Gascón, Joan-FrancescRicke-Hoch, MelanieFonseca, FernandoRiddick, Eric WellingtonFontes Ribeiro, CarlosRideout, HardyForero-Castro, MaribelRidola, LorenzoFormoso, ElenaRiefolo, MattiaFornalski, KrzysztofRiek, RolandForrer, FlavioRiemma, GaetanoForschner, AndreaRies, FrancisFort, IsabelRiethman, Harold C.Fortino, MariagraziaRiggio, OlivieroFošnarič, MihaRigillo, GiovannaFoulon, ChrisRinaudo, MarcoFowler, Elizabeth V.Rinschen, MarkusFox, SimonRitter, ManuelFox-Powell, MarkRiva, GiuseppeFracasso, GiulioRizzo, AngelaFrancesco, ScavelloRobert Jeyasingh, Samuel PushparajFranchi, CaterinaRobertson, PaulFranchi, ElisabettaRobles, Nicolas RobertoFrancis, Brian R.Rocha, CláudiaFrancis, Ian CRoche, PhilippeFrancisco Javier, Toledo-SolísRochfort, KeithFrancisco, InêsRočka, SauliusFrancisco-Morcillo, JavierRodrigo, GuillermoFranco, DomenicoRodrigues, Fernando José SantosFranco, Marina SantiagoRodríguez López, María IsabelFranco, PierfrancescoRodríguez, GuillermoFrançois, CasasRodriguez-Valera, FranciscoFrandes, MirelaRoels, JulietteFrank, KrisztiánRoghanian, MohammadFrankel, AdamRohaim, Mohammed A.Franzoni, FerdinandoRohn, SaschaFrasca, LoredanaRojo Tirado, Miguel ÁngelFraser, ClareRojo, Maria AngelesFrattini, FrancescoRola, PiotrFrattolin, JenniferRoldão, AntónioFrazier, Hilaree N.Rolla, GiovanniFreissinet, CarolineRolo, JoanaFrey, SilkeRomano, AngeloFribley, AndrewRomano, EloisaFrietze, Seth E.Romano, GiovannaFriščić, MajaRomanyuk, NataliyaFritsch, H.Rombolà, LauraFroelich, Matthias F.Romero, AlejandroFrolov, AndrejRomero-Masters, JamesFrontasyeva, MarinaRommel, FelixFroschauer, Stefan M.Romosan, Radu-StefanFrutos, RogerRondon, Ana G.Frutos, SergioRonjat, MichelFuertes-Guiró, FernandoRosa, Julia M.Fujii, KengoRosario, Fredrick J.Fujii, ShoRosati, AlessandraFujimura, AtsushiRose, PeterFujisawa, MasanoriRoseanu Constantinescu, AncaFujiwara, KeiRosendale, Andrew J.Fujiwara, KeigiRosenthal, KenFukami, KimioRoss, David S.Fukuda, MakihaRoss, Edward VictorFukui, KojiRostoker, GuyFukumoto, YoshihiroRothbauer, MarioFukunaga, TsukasaRothberg, PaulFukushima, AtsushiRoubelakis-Angelakis, Kalliopi A.Fumin, PanRoumeliotis, StefanosFuruya, KishioRouthu, Nanda KishoreFusi, JonathanRoveta, FaustoGabel, FrankRox, KatharinaGábor, BernátRoy, DhruvajyotiGabriel, Roldán-RoldánRoy, Swapan KumarGabryelska, AgataRozalski, MarcinGabryelska, MartaRozenfeld, Paula A.Gac, PawełRozenfeld, Paula AdrianaGackowski, DanielRozwadowska, NataliaGagniuc, PaulRuaro, BarbaraGala, Rikhav PRubanovich, AleksanderGalanis, AlexisRuben, PereiraGalanopoulos, MichalisRubi-Fessen, IlonaGalbiati, AndreaRubin, SebastienGalicka, AnnaRudert, MaximilianGaliero, RaffaeleRudkowska, IwonaGalindo, Cristi L.Ruf, AlexanderGallelli, LucaRuggiero, AlessandraGalletti, FrancescoRuggiero, AngeloGallo, GaetanoRuiz-Mirazo, KepaGallo, GiovanniRuscetti, MarcusGallo, MonicaRusmini, MartaGalluccio, MicheleRussell, MichaelGaman, Mihnea-AlexandruRusso, AlessandroGamberucci, AlessandraRusso, DomenicoGameiro, Paulo A.Rusu, Mugurel ConstantinGanesh Kumar, NishantRuvio, RaquelGanguly, PayalRuzza, PaoloGanguly, PritamRzepecka-Stojko, AnnaGannibal, PhilippSaad, AhmedGarami, AndrasSaavedra-Molina, AlfredoGarcia-Descalzo, LauraSaba, FakhredinGarcia-Jares, CarmenSabatino, LauraGarcía-Ríos, M. CarmenSabella, ErikaGarcía-Rodríguez, NéstorSabovljevic, MarkoGarcía-Romero, NoemíSack, Laura M.Garcia-Sherman, MelissaSadana, PrabodhGarcía-Silva, SusanaSaddala, Madhu SudhanaGarigliani, MutienSadhasivam, BalajiGarofolini, AlessandroSaeednejad Zanjani, LeiliGarrido, AdriánSagar, RamGarrido, EduardoSaghaeian Jazi, MarieGarstka, MaciejSagredo, OnintzaGaspar, KrisztianSaha, BedabrataGass, Justin T.Saha, RanajayGaur, PankajSahgal, PranshuGawda, PiotrSahu, Kamal KantGawęcki, MaciejSaini, ShikhaGazerani, ParisaSak, Jarosław J.Gazia, FrancescoSakai, MotokiGazzin, SilviaSakhno, YuriyGeladas, NickosSakurada, YoichiGelbenegger, GeorgSala, GessicaGelemanović, AndreaSalazar, EmelynGendron, NicolasSalehpour, FarzadGenet, CarineSalem, MounirGenovese, ClaudiaSalgado-Boquete, LauraGeorgakilas, AlexandrosSalladini, EdoardoGeorge, KoumantakisSalman, MootazGeorge, SantoshSalmanton-García, JonGeorgescu, Simona RoxanaSalvador, ArmindoGeorgiadi, AnastasiaSalvati, BrunoGeorgianos, Panagiotis I.Salvatore, SilviaGeorgousopoulou, Ekavi N.Samardžija Nenadov, DraganaGeppe, NataliaSamarzija, IvanaGerdol, MarcoSambri, AndreaGermanotta, MarcoŠamec, DunjaGesundheit, BenjaminSamiec, MarcinGhelardoni, SandraSampaio-Marques, BelémGhidini, MicheleSamujh, RamGhimire, Bimal KumarSánchez Quinteiro, PabloGhobadloo, ShahrokhSanchez-Alcazar, JoseGiacomelli, LucaSanchez-Martin, ManuelGiannakouros, ThomasSánchez-Rico, MarinaGiannella, LucaSandhu, KiranGianturco, LuigiSándor, Attila DGica, NicolaeSanguedolce, FrancescaGiganti, FrancescoSanjeewa, Kalu Kapuge AsankaGil, EricSanmukh, Swapnil GaneshGil-Calvo, MarinaSano, MakotoGiniger, EdwardSansone, AndreaGinsberg, Stephen D.Santana, Andrés GonzálezGioacchini, GiorgiaSantarpino, GiuseppeGiorgakis, EmmanouilSantella, BiagioGioti, KaterinaSanti, MelissaGippet, Jérôme M. W.Santos, Janete QuelhasGiraudo, Maria TeresaSantos, João H. P. M.Giron, LeilaSantos, João M.Gissot, ArnaudSapara, AdegboyegaGiuliani, Anna LisaSara, FranceschelliGiuseppe, ArcangeliSaraiva, MagdaGłąbska, DominikaSaraiva, Miguel MascarenhasGlogowska, AleksandraSaravanakumar, KandasamyGlotov, AndreySarecka-Hujar, BeataGlushkova, NatalyaSarkozy, MartaGnat, SebastianSarrigiannis, Ptolemaios GeorgiosGniazdowska, EwaSarul, MichałGocheva-Ilieva, Snezhana GeorgievaSato, HirokiGockel, Pancreatic Cancer CellsLukasSato, YasutoGodinho, CatarinaSatyamitra, MMGodos, JustynaSaurav, KumarGołąbek-Sulejczak, DorotaSavchenko, Tatyana V.Golan, DanielSavić, JelenaGoldenkova-Pavlova, IrinaSavidor, AlonGolec, PiotrSavira, FebyGolestaneh, NadySavoia, PaolaGolomazou, EleniSavoie, JeanGolovics, Petra AnnaSavransky, VladimirGómez Castilla, JordiSawada, Jun-ichiGómez, F.Sawant, AkshadaGómez-García, Francisco JoséSawayama, EitaroGómez-Manzo, SaúlSawicki, RafalGómez-Martín, BeatrizScacco, SalvatoreGomzikova, MarinaSchaaf, Gerben J.Gonçalves, Ana CristinaSchaafsma, A.Gonzalez, Diego LuisSchaefer, JochenGonzalez, GabrielSchantz, Susan L.González, JerónimoSchatten, HeideGonzález-Andrade, MartínSchcolnik-Cabrera, AlejandroGonzález-Granado, Luis IgnacioScher, Mark S.González-López, Marcos A.Scherma, MariaGonzalez-Menendez, PedroSchiattarella, GabrieleGonzalez-Rodriguez, AlexandreSchiffer, EduardoGopinath, DivyaSchildkraut, CarlGóra, Robert W.Schippa, SerenaGordo, IsabelSchläfli, Anna M.Goričar, KatjaSchloer, SebastianGornowicz-Porowska, JustynaSchloss, JanetGorny, AdrienneSchmaier, Alvin HGorres, Josef H.Schmidt, BarbaraGoto, KatsumasaSchmitt, FelixGovender, LaurenciaSchnider, ArminGovernini, LauraSchroer, Martin A.Grabarek, Beniamin OskarSchützenberger, AnneGrabovac, IgorSchwarz, KonstantinGrade, SofiaSchwarzer, RolandGrafi, GideonSchwenkenbecher, PhilippGraille, MarcScibior, AgnieszkaGranato, MarisaScolieri, PalmaGrau, James W.Scrano, LauraGray, Nora ESeano, GiorgioGreco, AntonioSees, Julieanne P.Greco, NicolaSegal, GadGreen, StefanSegovia, CristinaGriggs, Katy E.Segu, MarziaGrigorenko, ElenaSeitlinger, JosephGrillo, AndreaSelby-Pham, JamieGrippi, FrancescaSelim, Abdelfattah M.Grishkan, IsabellaSelim, KhaledGrossbach, AndrewSelvarajan, EthirajGrosso, ClaraSelzer, LisaGrunow, BiankaSemova, IvanaGruschus, JamesSenel, MakbuleGrygorczyk, RyszardSeow, Kok-MinGrygorieva, OlgaSerban, DragosGryszczyńska, BognaSeret, BernardGrzegorczyk, IzabelaSereti, EvangeliaGu, BinSermyagin, Alexander A.Gu, LinxiaSerralheiro, Maria LuisaGu, SiyiSerrano-Sánchez, María De LourdesGuagliardi, IlariaSessa, FrancescoGuardado, IsmaelSestras, Adriana F.Guardia, CarlosSethi, GautamGudsoorkar, PrakashSethi, ManveenGueguen, NaïgSeto, ToshiyukiGuerra, Alfredo J.Sever, BelginGuerra, FloraSevillano, ÁngelGuerra-Castellano, AlejandraSeyedAlinaghi, SeyedAhmadGuerrero, Sandra M. MartinSfeatcu, Ionela RuxandraGuerriero, IlariaShaffery, JamesGuglielmo, PriscillaShaik, Haq AbdulGuidi, PatriziaShalaby, Mohammed NaderGuidotti, GiuliaShamshiri, Redmond RaminGuiliani, NicolasShane, EnglishGuillard, MaryShao, LijianGuillen-Grima, FranciscoShao, Yu-Hsuan JoniGull, MaheenShapiro, JamesGunderson, Mark P.Sharafutdinov, IrshadGünes-Bayir, AyseSharma, AmitGuo, LilongSharma, AnirudhGupta, SanjeevSharma, DivyaGureev, VladimirSharma, GauravGurgul, ArturSharma, NidhiGursky, VitalySharma, PiyushGut, PawełSharma, RohanGutekunst, KirstinSharma, ShrikantGuyot, RomainSharmila V, GodvinGuzmán, José MiguelSharon, GalitGwon, Dong IlSharpe, ShaunGyöri, David S.Shavarda, AlexeyHafer, CarstenShaw, SophieHafiane, AnouarShek, DmitriiHafizi, SinaShen, JiangchuanHagihira, SatoshiShen, MengxiHaines, Julie ReiszShen, ShichenHajra, AdrijaSheorey, HarshaHakimiha, NedaSherman, MichaelHalperin, John JSheval, EugeneHamazaki, JunShiga, KiyotoHamelin, JérômeShimakawa, GingaHamidi, OksanaShimizu, KazuyukiHammer, Bruce E.Shin, SooimHammer, MartinShindo, YutakaHammerl, Jens AndréShingu, YasushigeHammouda, Manel BenShinichi, GotoHamon, MorganShirahata, TatsuyaHampson, LynneShirole, NitinHan, ChunleiShiue, Yow-LingHan, DongShnayder, Natalya A.Hang, PengzhouShopova, DobromiraHano, ChristopheShoshkes-Carmel, MichalHansíková, HanaSiaw, JoachimHao, JihuaSiddiqui, ArifHaragus, HoriaSiebert, Hans-ChristianHarasti, DavidSieradzan, AdamHarris, LarrySierra, Josep M.Harris, Rachel L.Sillanpää, NikoHart, MelanieSillerud, Laurel O.Hartman, MariuszSilva, Ana FilipaHasan, MohammadSilva, ElisabeteHasegawa, MakotoSilvestris, NicolaHashem, NesreenSimionescu, AncaHasnagas, Nikolaos D.Simitzis, PanagiotisHassan, MuhammadSimkus, DanielleHazama, ShyouichiSimon, LukeHazrati, Lili-NazSimonsen, Anja HviidHe, LiangcanSinai, Anthony P.He, MingyuŠindlerová, LenkaHe, ShaoqiuSinescu, Ruxandra DianaHeaton, Steven M.Singh, Abhishek KumarHedman, HaydenSingh, AkankshaHeidor, RenatoSingh, KavitaHeldin, ParaskeviSingla, RajeevHellmann, NadjaSinha, DivyaHellström, StenSiniscalco, DarioHelm, SabrinaŠípek, PetrHendrik, BargelSipos, FerencHenk, Daniel A.Siristatidis, CharalamposHenkel, ChristiaanSiriyappagouder, PrabhugoudaHenrich, DirkSisoeva, OlgaHenriques, Ana RitaSitnikov, DmitryHenriques, JoaquimSkarżyńska, EwaHenrot, PaulineSkopinska-Wisniewska, JoannaHepojoki, SatuŠkrlec, IvanaHernández-Hernández, OscarSkrypnik, KatarzynaHerrick, Sarah ElizabethSkuja, SandraHeyer, RobertSkuza, LidiaHibbard, PaulŚlaska, BrygidaHickman, SimonS.M. Jawad, AliHickman-Lewis, KeyronSmith, AlanHiggs, PaulSmith, Caroline J.Hildebrand, JoannaSmith, EricHirade, TomohiroSmith, J. KellyHirono, KeiichiSmith, Micholas DeanHiroyuki, KobayashiSmonou, IouliaHo CM, RogerSobočan, MonikaHo, Jar-YiSobotta, ŁukaszHofer, EdithSocea, BogdanHöfer, KatharinaSocolov, RazvanHolb, ImreSodano, FedericaHolcman, KatarzynaSoderhall, IreneHolleran, Katherine MercedesSogawa, NorioHolloway, David M.Sohail, MuhammadHolm, Nils G.Soica, CodrutaHolt, Stephen A.Sokolovskaya, AlisaHolzhauer, MennoSokołowski, AndrzejHolzmann, KlausSoldovieri, Maria VirginiaHomma, KoheiSolé Cañadas, CarlaHong, BoohwiSominskaya, IrinaHong, Chi-eunSon, Young-OkHong, Sung NohSongsasen, NucharinHorai, YoshiroSophocleous, Reece AndrewHorecka, BeataSoto, Esther CuadradoHorev, AnatSoule, TanyaHori, MikaSousa, AngelaHormigo, SebastiánSousa, KeissyHorváth, ÁdámSousounis, KonstantinosHorváth, Eszter MáriaSouza, Divanizia Do NascimentoHou, ChihYaoSpadaro, AngeloHoughton, Conor J.Sparagna, Genevieve C.House, Christopher H.Spasic, MiodragHouston, BrendanSpeciale, ImmacolataHoving, EelcoSpencer, Rebecca N.Hrenak, JaroslavSpielmann, TobiasHrnčić, DraganSpiess, Andrej-NikolaiHsia, Shih-MinSpitaleri, AndreaHsu, Chun-ChunSpletter, MariaHsu, Te-HuaSponer, JuditHsu, Yu-ChiaSquassina, AlessioHuang, Cheng-YangSquizzato, AlessandroHuang, Chia-ChiSrivastava, AmitHuang, Eng-YenSroubek, JanHuang, HsiyuanStamatakis, KonstantinosHuang, Hui-ChiStanciu, Gabriela-DumitritaHuang, Jian ZhiStaniek, HalinaHuang, RongStanojevic, Dragana M.Huang, TaoStanzione, ArnaldoHuet, EricStarowicz, MałgorzataHuh, Joo YoungStătescu, CristianHulme, JohnStausholm, Martin BjørnHumphries, BrockStchigel, Alberto MiguelHupalo, KamilStecco, CarlaHur, HoonSteemans, PhilippeHussain, MulazimStefano, AlessandroHuth, SebastianSteiger, AxelHutorowicz, AndrzejStepanova, Anna A.Hwang, Candy SStepien, Karolina M.Hwang, WonjungStępniewski, JacekIchihashi, NorikazuŠtern, AljaIchikawa, TomohiroStewart, AdeleIdo, AtsushiStimpfel, MartinIfergan, IgalStines-Chaumeil, ClaireIglesias-Soler, EliseoStöcker, FabianIkehara, KenjiStoica, MaricicaIles, GailStojanov, MilosIlinskiy, YuriyStojković, DejanIlyin, AlexeyStomeo, FrancescoImbriano, CarolStone, WendyImmohr, Moritz BenjaminStoyanov, DrozdstoyIndino, CristianStrauß, MarkusIndra, RadekStrbak, OliverIngham, Colin J.Streel, MauriceIngram, Krista K.Strenzke, NicolaInoue, DaisukeStriker, Rob T.Invitto, SaraStrycharz, JustynaIoannou, LeonidasStrzalkowski, NickIolascon, GiovanniStrzelecki, DominikIosif, PediaditakisStuckey, RuthIosifidis, MichaelSu, YajuanIovino, DomenicoSubbiahdoss, GuruprakashIrakli, MariaSubbotin, Sergei A.Isaeva, Marina P.Subirana, JuanIshii, ShunichiSubramanian, PremIshikawa, TetsuoSuda, GokiIslam, MonirulSuenaga, MitsukuniItabashi, MichioȘuică-Bunghez, RalucaItami, JunSułkowska, MałgorzataIto, EtsuroSullivan, Genevieve MaryIto, NaokiSuman, ShubhankarIvanišević, AnaSun, JingIvanova, SaskaSundararajan, KousikIvanovska, NinaSundararajan, RajiIvastinovic, DomagojSuntornnond, RatimaIwamuro, MasayaSuravajhala, PrashanthIwata, HiroakiSuriya Prakash, GanesanIwata, KentaroSurowiak, PawełIzawa, Kazuhiro P.Suter, AndyIzumi, KojiSuzuki, KazuhitoJaburek, MartinSuzuma, K.Jachimska, BarbaraSvegliati, SilviaJackova, AnnaSvetlik, JanJackson, RobertŚwitońska, MilenaJacob, SaritaSynowiec, AgnieszkaJacquelin, SebastienSzczepanik, DariuszJafarnejad, Seyed MehdiSzczubialka, KrzysztofJagga, SupriyaSzerement, JustynaJakobsson, MagnusSzewczyk, KatarzynaJames, JoelSzewczyk, Nathaniel J.Jamka, MałgorzataSzilák, LászlóJamroz-Wis̈niewska, AnnaSzymanska, MagdalenaJamshidi, MohammadSzymańska, MagdalenaJamy, Omer HassanSzymczak-Pajor, IzabelaJanda, TiborTaanman, Jan-WillemJaneček, ŠtefanTaiar, RedhaJanik, MiroslawTakabatake, KiyofumiJaniszewski, PiotrTakahashi, AkihisaJanowska, AgataTakashima, ShigeoJanssen, Maarten P.F.Takeno, MitsuhiroJanuel, EdouardTalukdar, SarmisthaJao, Tzu-MingTamayo-Ortiz, MarcelaJaric, IvanaTamir, Sharon OvnatJaromír, LukavskýTampe, BjörnJas, Gouri S.Tamura, KojiJash, BiswarupTan, Loh Teng-HernJasińska, Karolina D.Tanabe, ShihoriJawien, JacekTanaka, MasaruJazirehi, Ali R.Tanaka, RyouJazmati, DannyTaniguchi, MasaakiJee, YongseokTanzer, MichaelJellen, RickTao, JunyanJeng, Geng-ShiTarantino, DomizianoJeong, Dong-HoonTarantino, GiuseppeJevremovic, SladjanaTarantino, NicolaJheeta, SohanTaratkin, Mark SergeevichJiang, LinTardáguila-García, AroaJiang, YongTaubner, Ruth-SophieJibin, ZhangTaylor, Mark S.Jimenez-Perez, IreneTebbe, Johannes J.Jimenez-Rejano, José-JesúsTeisseyre, AndrzejJiří, PatokaTerrinoni, AlessandroJirkovsky, EduardTerry, CassandraJo, SungsinTerry, DanielJohansson, LindaTessera, MarcJohnston, BrianTestarelli, LucaJohnstone, CameronTeufelberger, Andrea R.Jomaa, Mona K.Texido Bartes, RobertJones, DarrylThanigaimani, ShivshankarJong, Chian JuThekkiniath, JoseJoniec, JolantaTheodorou, John A.Joshi, GauravThiem, Daniel G. E.Juárez-Flores, Diana LuzThirion, Daniel J.G.Juif, Pierre EricThompson, KatharineJuiz-Valiña, PaulaThorn, CarolineJuliusson, GunnarTian, SonghaiJung, Jin-HwaTidy, AlisonJung, Ki JinTimofeeva, Angelika V.Jung, Man-YoungTirard, StephaneJung, SungmokTirtei, ElisaJung-Klawitter, SabineTlotleng, NonhlanhlaJuszczak, Grzegorz R.Toczek, JakubKabisch, StefanTodorov, SvetoslavKacar, BetulTogawa, AtsushiKacergyte, InetaTomaszkiewicz, MartaKadokawa, Jun-ichiTommasin, SilviaKagaya, YuTondo, GiacomoKai, LeiTorlakovic, Emina EmiliaKainz, HansTornesello, AssuntaKajjumba, George WilliamTöröcsik, DánielKajtoch, ŁukaszTorras-Ambròs, JoanKakegawa, TakeshiTorres Vaamonde, José EnriqueKalainayakan, Sarada PreetaToth, DenesKalas, WojciechTouloupakis, EleftheriosKalinowski, Brenton D.Tramonti, AngelaKalofonou, FoteiniTrapp, OliverKalyvas, Aristotelis VTravassos, Denise VieiraKalyvas, NektariosTrcek, JanjaKameya, MasafumiTrentanovi, GiovanniKamyab, HesamTrimarco, BrunoKanczler, Janos M.Tripathi, RakshamaniKandimalla, RameshTripathi, Satyendra C.Kaneko, GenTroan, Brigid VictoriaKang, EunjuTrovato, MariaKantor, AsherTsai, Kun-LinKao, Cheng-YenTsai, Ming-HorngKapazoglou, AlikiTsai, Ming-JuKaplan, PeterTsai, RongkungKar, AnirbanTsakmakidis, Ioannis A.Kar, SouvikTse, William K.F.Karaduta, OlegTseng, PhilipKarahan, Ali YavuzTsermoulas, GeorgiosKarakitsos, DimitriosTsubota, AkihitoKaranis, PanagiotisTu, Anthony T.Karatzanos, EleftheriosTubić, AleksandraKarczmarska-Wódzka, AleksandraTudek, AgnieszkaKarobari, Mohmed IsaqaliTun, Han NaungKarpienko, KatarzynaTvrdik, PetrKarpov, DmitryTylki-Szymańska, AnnaKarsten, Christina B.Tzeng, I-ShiangKarwowski, BoleslawTzeng, Nian-ShengKasak, LauraUchimura, KoheiKasarda, RadovanUddin, ShahabKashi, AmitUetrecht, CharlotteKashiwagi, AkikoUgon, AdrienKashiwagi, ShinichiroUllah, Mohammad FahadKashtalap, VasilyUlusu, NurayKastaniotis, Alexander J.Ulz, PeterKataoka, HiroshiUmbarkar, PrachiKato, YasuhikoUndersander, DanKatris, NicholasUnger, Lukas W.Kaushik, Nagendra KumarUngureanu, Bogdan SilviuKaushik, ShubhiUntergasser, AndreasKawakami, JunjiUppugunduri, Chakradhara RaoKaznina, NataliaUrbaniak, CamillaKazunori, MatsudaUrbańska, Ewa M.Kazunori, TomitaUrgel, Manuel EspinosaKebukawa, YokoUziel, OritKeller, Jonathan R.Vaca-González, Juan JairoKemper, BjörnVaira, Luigi AngeloKen, InoueValan Arasu, MariadhasKeniry, AndrewVale, NunoKenji, ToyotaValenti, DanielaKeogh, KateValenti, PaolaKereszturi, AkosValentino, MarioKermani, Abbas JafariValvo, Paolo SebastianoKhalil, Ahmed Mohamed AliVamvakaris, IoannisKhatib, SolimanVan Den Ameele, JelleKhaw, Kooi YepngVan Den Berg, BernardKieliszek, MarekVan Der Westhuizen, Emma T.Kiga, Daisukevan Gammeren, Adriaan J.Kim, BoyongVan Grevenynghe, JulienKim, Chang-BaeVan Haren, Simon D.Kim, Cuk-SeongVan Loon, Bert Jan PotterKim, DennisVanegas, JairoKim, Dong-YeopVania, AndreaKim, Ga-YeongVannier, Jean-BaptisteKim, Gheun-HoVardasca, RicardoKim, GuoweiVarela, GonzaloKim, Hong-DuckVarrella, StefanoKim, Hyeong-GeugVasaitis, RimvysKim, HyojoongVasconcelos, Elton J. R.Kim, Hyoun-AhVasiliadou, RafaelaKim, Hyun-JunVassilieva, Galina YuKim, JeongheeVasudevan, PraveenKim, Jin JuVaux, FelixKim, JinhaeVaze, Nachiket D.Kim, JongminVázquez-Marrufo, GerardoKim, KijinVecchio, DomiziaKim, Kil-SooVelada, IsabelKim, Kyoung SooVelayutham, MurugesanKim, LyndonVélez, Emilio J.Kim, Mi-KyungVella, Filomena MonicaKim, Sung HanVenâncio, CátiaKim, SungminVenditti, FrancescoKim, TaewonVengoji, RaghupathyKimura, SatoshiVenkatachalam, MekalaKinoshita, AkihikoVenkatadri, RajkumarKinspergher, StefaniaVenter, Stephanus N.Kioshima, Erika SekiVenturelli, MassimoKirsch, Alexander HVerde, FedericoKishita, YoshihitoVergadi, EleniKisielewicz, KamilVernuccio, FedericaKiss, AttilaVerseux, CyprienKiss, John Z.Verykouki, EleniKitamura, YoshiakiVesa, Stefan CristianKleczka, AnnaViana, FlaviaKlee, SaschaViazis, NikosKleeff, JörgViazzi, FrancescaKlimontov, VadimVicaș, Laura GrațielaKlimova, BlankaVicente, DaviniaKłodawska, KingaVicente-Manzanares, MiguelKloss-Brandstätter, AnitaVieira, PedroKlutsch, CornelyaViel, GuidoKnapic, SofiaViennet, ElvinaKo, Chih-HungVigh, JozsefKo, Chiung-YuanVijayan, KamalakannanKo, Kyung RaeVilla, ChiaraKobeissy, FirasVillalain, CeciliaKoblmüller, StephanVilla-Ruano, NemesioKodziszewska, KatarzynaVillasante, AranzazuKoh, Jin-HoVillena, JuanKohlmann, KlausVilović, MarinoKoksharova, Olga A.Vincent, AmyKolář, MilanVishnyakov, Innokentii E.Kolbasov, DenisVismara, ElenaKolikonda, Murali KrishnanVitiello, DamienKolmar, HaraldVitiello, LivioKolodziejczyk, AleksandraVlk, MartinKoltz, Michael T.Vodnala, MunenderKomati, RajeshVogel, MatthiasKomatsu, HidetoshiVogelsang, HaraldKomlenac, NikolaVogiatzaki, ChrysanthiKomori, AtsumasaVohník, MartinKong, Pui WahVoiculescu, DanKonieczny, PatrykVolpicelli, FlorianaKönig, GerhardVorkas, Charles KyriakosKonno, NaotakeVorobyev, ArtemKopylov, ArthurVos, MelissaKorfitis, ChrysovalantisVoskarides, KonstantinosKorivi, MallikharjunVoulgarelis, MichaelKormutak, AndrejVranes, MilanKosakivska, Iryna V.Vrentas, Catherine E.Kosinski, JanVujicic, MiloradKostas-Agnantis, Ioannis P.Vydra, NataliaKostić, Aleksandar Ž.Wagner, Kay-DietrichKostylev, Pavel I.Wahab, RizwanKostyuchenko, RomanWajnberg, GabrielKostyushev, DmitryWalczak-Drzewiecka, AureliaKotaška, KarelWalczak-Jędrzejowska, RenataKotchi, Serge-olivierWaldhelm, AndyKotenkova, ElenaWaldman, MaayanKotula-Balak, MałgorzataWalicka-cuprys, KatarzynaKoubassova, NataliaWan, LeiKouidi, EvangeliaWang, Aline Yen LingKoutelidakis, IoannisWang, AnnaKovács, BélaWang, ChihyangKovács, Viktória AnnaWang, HongwuKovarik, AlesWang, HsiuyingKovtun, OlegWang, HuiKowalska, KarolinaWang, JiangxinKoyama, SachikoWang, Jing J.Kozak, JoannaWang, MaosenKozieł, MonikaWant, Muzamil YaqubKrafft, Frieder C.Warnecke, GregorKrajnak, Kristine M.Warzecha, ZygmuntKraus, ArminWasniewska, MalgorzataKrawczyk, BeataWatanabe, EiichiKrawczyk, PrzemekWatanabe, KazuyukiKresoja-Rakic, JelenaWatkins, JamesKrier, FrançoisWatterson, Daniel MartinKrishnamachary, BalajiWatts, Phillip C.Krockow, EvaWawruch, MartinKrokidis, Marios G.Wawrzkiewicz, MonikaKrujatz, FelixWebb, ClintonKrulová, MagdalénaWee, Sang OukKrysta, KrzysztofWei, JinKrzysiak, Michał K.Weigmann, SimonKubiak, Magdalena ReginaWeinzierl, RobertKubínová, RenataWeiser, LukasKucharczyk, DariuszWeiss, Irwin K.Kucharski, MariuszWeissensteiner, HansiKujawska, MałgorzataWerth, Alexander J.Kuki, ÁkosWestall, FrancesKuliczkowski, KazimierzWestenskow, PeterKülköylüoğlu, OkanWhitman, MaryKumagai, ShinyaWidemann, EmilieKumakura, SatoshiWięch, PawełKumar, AjayWiedemann, JuliaKumar, HirdeshWierzbicka, ElżbietaKumar, JananiWiesik-Szewczyk, EwaKumar, KishoreWigner, PaulinaKumar, LokeshWilke, DiegoKumar, ManuWilkinson, RobKumar, RavindraWilkowska, AlinaKumar, RupeshWillmore, William G.Kun, ÁdámWilson, Jamie L.Kunert, RenateWilson, Jean M.Kunnev, DimiterWitalisz-Siepracka, AgnieszkaKuo, Mei-ChuanWitt, BarbaraKurg, ReetWłodarczyk-Stasiak, MarzenaKuroda, HidekatsuWong, Albert S. Y.Kuroi, YasuhiroWong, Antonio CPKurtenbach, StefanWong, Chi-MingKurukulaaratchy, Ramesh JWong, Chin-CheanKuruma, YutetsuWong, Season S.Kuryk, LukaszWörtwein, GittaKuryłowicz, AlinaWu, ChaoKusamori, KosukeWu, Chen-ChiKušan, IvanaWu, QihuiKusztal, MariuszWu, WeiKutikhin, Anton G.Wu, YinoKwack, Mi-heeWüest, SimonKwan, Mike YwWunder, ChristianKwon, Hyung JooWyganowska-Świątkowska, MarzenaLa Penna, GiovanniWylęgała, EdwardLa Rocca, Renato V.Xi, JinxiangLaboisse, Christian L.Xiong, LanLabuda, RomanXu, LiyaLabudda, MateuszXue, FengLabuz-Roszak, BeataYadav, DhananjayLacabanne, DenisYamamoto, NaokiLadoukakis, ManolisYamasaki, MasaoLafont, ElodieYang, Jee MyungŁagowska, KarolinaYang, JinyanLai, Ching TatYang, LetaoLai, Wen-FuYang, Wen-BinLakicevic, NemanjaYao, ChangfuLal, AmosYasuda, HiroshiLalitkumar, Parameswaran GraceYen, Cheng-FangLall, Gurprit S.Yoder, Karmen K.Lamango, Nazarius S.Yoo, JaeryongLambova, SevdalinaYoshioka, ShinichiLan, ShenghueiYu, JeongilLancel, SteveYu, QingLandi, LuciaZabucchi, GiulianoLange, Maren D.Zacchia, MiriamLanza, GiuseppeZahan, MariusLapucci, AndreaŽaja, OrjenaLaranjo, MafaldaZakaria, Basem S.Larionova, IrinaZakłos-Szyda, MalgorzataLarson, Steven RZakrocka, IzabelaLasecka-Dykes, LidiaZalevsky, ArthurLaselva, OnofrioZalewski, Daniel P.Lasseur, ChristopheZamorano-Leon, José JavierLatosinska, AgnieszkaZamudio, Gabriel S.Lattanzi, SimonaZanetti, DiegoLaura, Lafon-HughesZaninotto, Martina M.Laurentia, AlexandrescuZapała, ŁukaszLauridsen, Jørgen TrankjærZardo, GiuseppeLauzon-Joset, Jean-FrancoisZarebidoki, AlirezaLavano, AngeloZarębska-Michaluk, DorotaLavender, AndrewZasina, DamianLavogina, DarjaZaslavsky, BorisLawrence, David A.Zatulovskiy, EvgenyLayer, LilianaZaucke, FrankLazare, SekaZavattaro, ElisaLe Merrer, JulieZawieja, IwonaLe, Nguyen Quoc KhanhZdravković, MarkoLeal-Junior, Ernesto Cesar PintoZdzisińska, BarbaraLeal-Marin, SaraZec, NebojšaLebert, MichaelZelger, BernhardLebiedzinska-Arciszewska, MagdalenaZeng, BiaoLee, Bu-kyuZerbo, StefaniaLee, Chang-MinZhao, LinlinLee, Chien-TeZhao, NingLee, Cho-HaoZhdankina, Yuliya S.Lee, Hsin-ChenZheng, ZhongLee, HyungseokZhiponova, MiroslavaLee, JieZhong, ChenhanLee, K.M.-C.Zhou, PingLee, Matthew Meng YangZibar, LadaLee, MichaelZiegler-Devin, IsabelleLee, Sang-HanZielinski, MarcinLee, SewonZipperle, JohannesLee, Sonny T.M.Zmijewska, AgataLee, SookyungZouboulis, Christos C.Lee, Soong DeokZuk, PiotrLee, Wei-ChiehZuo, HaoLee, WoojooZydney, AndrewLehmann, ChristianZyśk, Marlena


